# Impact of body mass index on worsening of diastolic function and impairment of left atrial strain in the general female urban population: a subanalysis of the Berlin female risk evaluation echocardiography follow-up study

**DOI:** 10.3389/fcvm.2023.1242805

**Published:** 2023-09-20

**Authors:** Elena Romero Dorta, Adrian Wolf, Anne Hübscher, Daniela Blaschke-Waluga, Ute Seeland, Claudia Crayen, Sven Bischoff, Isabel Mattig, Henryk Dreger, Karl Stangl, Vera Regitz-Zagrosek, Ulf Landmesser, Fabian Knebel, Verena Stangl, Anna Brand

**Affiliations:** ^1^ Deutsches Herzzentrum der Charité, Department of Cardiology, Angiology and Intensive Care Medicine, Campus Mitte, Berlin, Germany; ^2^ Charité—Universitätsmedizin Berlin, Corporate Member of Freie Universität Berlin and Humboldt-Universität zu Berlin, Berlin, Germany; ^3^ DZHK (German Centre for Cardiovascular Research), Partner Site, Berlin, Germany; ^4^ Institute of Social Medicine, Epidemiology and Health Economics, Charité—Universitätsmedizin Berlin, Corporate Member of Freie Universität Berlin and Humboldt-Universität zu Berlin, Berlin, Germany; ^5^ Freie Universität Berlin, Department of Education and Psychology, Berlin, Germany; ^6^ Berlin Institute of Health at Charité—Universitätsmedizin Berlin, BIH Biomedical Innovation Academy, Berlin, Germany; ^7^ Charité—Universitätsmedizin Berlin, Institute of Gender in Medicine, Berlin, Germany; ^8^ Department of Cardiology, University Hospital ZüRich, University of ZüRich, Switzerland; ^9^ Deutsches Herzzentrum der Charité, Department of Cardiology, Angiology and Intensive Care Medicine, Campus Benjamin Franklin, Berlin, Germany; ^10^ Clinical Department of Cardiology, Internal Medicine II, Sana Klinikum Berlin-Lichtenberg, Germany

**Keywords:** body mass index, left atrial strain, diastolic dysfunction, left atrium, BEFRI

## Abstract

**Background:**

The association of body mass index (BMI) with diastolic dysfunction (DD) is well described in the literature. However, there is conflicting evidence and long-term follow-up data regarding effects of BMI on preclinical DD and left atrial (LA) function are scarce, highlighting the importance of early detection tools, such as myocardial strain.

**Purpose:**

The aim of our study was to prospectively analyze the impact of clinical and demographic parameters, especially of BMI, on worsening of diastolic function and left atrial strain (LAS) in an urban population of women with a low prevalence of cardiovascular risk factors.

**Methods and Results:**

An extensive clinical and echocardiographic assessment comprising the analysis of phasic LAS using two-dimensional speckle-tracking echocardiography (2D STE) was performed in 258 participants of the Berlin Female Risk Evaluation (BEFRI) trial between October 2019 and December 2020 after a mean follow-up period of 6.8 years. We compared clinical and echocardiographic parameters stratifying women by BMI < or ≥25 kg/m^2^, and we analyzed the impact of demographic characteristics on the worsening of DD and LA mechanics in the longer-term follow-up using univariate and multivariate regression analyses. 248 women were suitable for echocardiographic analysis of LAS using 2D STE. After a mean follow-up time of 6.8 years, LA reservoir strain (LASr) and LA conduit strain (LAScd) were significantly reduced in participants with a BMI ≥25 kg/m^2^ compared with women with a BMI <25 kg/m^2^ at baseline (30 ± 8% vs. 38 ± 9%, *p* < 0.0001; −14 ± 7% vs. −22 ± 8%, *p* < 0.0001). 28% of the overweighted women presented a deterioration of diastolic function at the time of follow-up in contrast with only 7% of the group with a BMI <25 kg/m^2^ (*p* < 0.0001). BMI remained significantly associated with LAS reductions after adjustment for other risk factors in multivariate regression analyses.

**Conclusion:**

Overweight and obesity are related to impaired LAS and to a worsening of diastolic function after a long-term follow-up in a cohort of randomly selected women.

## 1. Introduction

Overweight and obesity have been linked to a worse diastolic function and an increased risk of heart failure and atrial fibrillation (1, 2). However, conflicting data suggest that there is no significant correlation between higher body mass index (BMI) and left ventricular (LV) diastolic dysfunction (DD) (3), and even a paradoxical protective effect of overweight and obesity has been discussed (4).

LA dysfunction is increasingly acknowledged for playing a main role in cardiovascular disease (5). Particularly LA function measured by phasic two-dimensional strain has shown its importance in diagnosing LVDD, especially in its early stages, and in grading its severity (6–8). Furthermore, including the analysis of LA strain (LAS) using two-dimensional speckle tracking echocardiography (2D STE) into a multimodality imaging algorithm seems to add important diagnostic value to the field of heart failure with preserved ejection fraction (HFpEF) (9), also in terms of affirming its specific aetiologies (10).

The effects of BMI on LAS have been described in a population of apparently healthy volunteers (11) highlighting the importance of early detection tools, such as myocardial strain. For the application of LAS in clinical practice, it is important to understand which factors determine the magnitude of its different phasic components. Therefore, setting the focus on BMI, we aimed to analyze clinical and demographic features associated with the natural course of evolving LVDD and LAS reduction and their deterioration in the mid-term follow-up in a population of randomly selected women.

## 2. Methods

### 2.1. General study population

The characteristics and design of the Berlin Female Risk Evaluation (BEFRI) trial have been already described (12). Briefly, the cross-sectional study included a randomized population of 1,066 women aged between 25 and 74 years of the city of Berlin, Germany, and aimed to analyze predictors of an incorrect cardiovascular risk perception in women. An extensive assessment of medical history including medication, conduct of somatometric measures, blood pressure measurement and ECG analysis were performed. Of the 1,066 participants, 473 women received a comprehensive transthoracic echocardiography during the recruitment in 2013 and 2014 (baseline), with a focus on DD assessment as well as analysis of myocardial LA strain. A detailed description of the echocardiographic data, setting the focus on left ventricular (LV) DD and LA strain, was first published after index investigations in 2016 (13). Somatometric data and clinical features of the larger sample at baseline have already been reported in detail (12). Every woman who took part in the BEFRI echo study and was suitable for the analysis of LA structure and function was invited for follow-up examinations between October 2019 and December 2020.

The trial was approved by the institutional ethics committee of the Charité University of Berlin (EA/2085/19), and all participants signed informed written consent.

For assessing the impact of BMI compared to other cardiovascular risk factors on LA function and DD, we defined two groups, following the World Health Organization (WHO) classification: a BMI <25 kg/m^2^ (normal) and ≥25 kg/m^2^ (overweight and obese).

### 2.2. Echocardiography

A comprehensive transthoracic echocardiography was conducted using a Vivid E9 system (GE Vingmed, Horton, Norway) with an M5S 1.5 to 4.5 MHz transducer, the same system used for baseline examinations. Standard echocardiographic views and measurements were performed in agreement with both, the American Society of Echocardiography (ASE) guidelines and the European Association of Cardiovascular Imaging (EACVI) guidelines (14, 15). We assessed diastolic function using established parameters as LA volume index (LAVI); diastolic transmitral inflow velocities (early—E wave—and late—A wave) and deceleration time obtained from the pulsed waved-Doppler signal; the lateral, septal and the average early diastolic mitral annular velocity (e') derived from the pulsed-wave tissue Doppler; and E/e'. LAVI was measured by the biplane disk summation technique in apical 4- and 2-chamber views. The maximum transvalvular velocity of the tricuspid regurgitation during systole allowed us to estimate the right ventricular (RV) to right atrial (RA) pressure difference. Structural and functional left and right heart parameters were then evaluated, as previously reported (13).

### 2.3. Analysis of left atrial function

**Figure 1:**
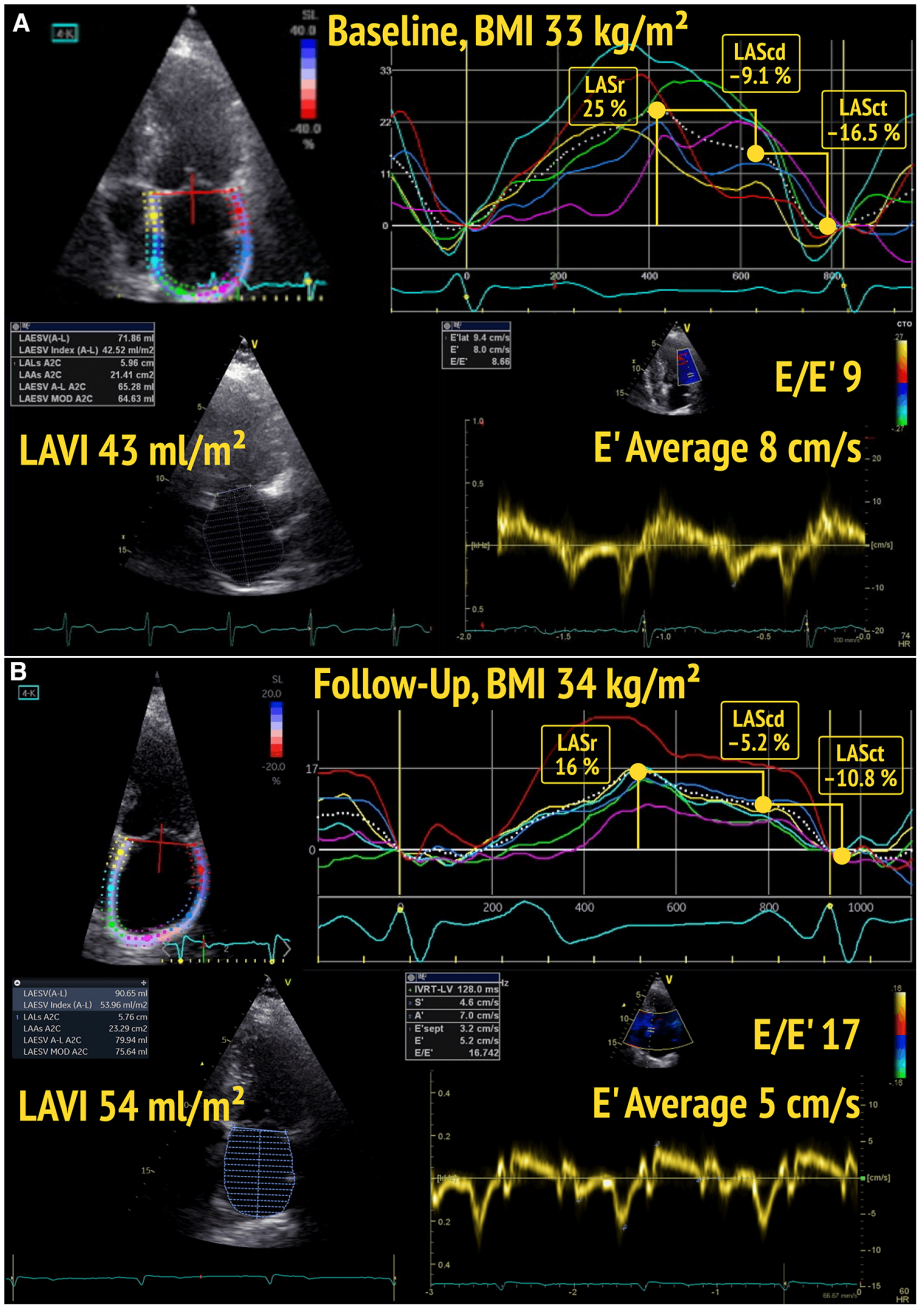
Baseline (**A**) and follow-up (**B**) echocardiography of a participant with BMI ≥25 kg/m^2^, showing a decline of left atrial reservoir strain (LASr) and left atrial conduit strain (LAScd), as well as a deterioration of diastolic function (from DD1 to DD2) over time.

Phasic LA function was assessed offline using 2D STE (EchoPAC v203, GE Healthcare) following the recent recommendations of the EACVI (16) and taking into account important technical and practical issues about image acquisition and post-processing (17). The analysis was performed in the LA focused apical 4-chamber view, avoiding foreshortening, and optimizing gain, depth, and frame rate (60–80 frames/s). Three cardiac cycles were recorded for each view. Since dedicated LAS software packages were not available in 2019 and 2020, and with the aim of following the methodology used in 2013 and 2014, the region of interest (ROI) was determined semi-automatically after tracing the endocardial borders of the LA using dedicated LV software. We analyzed LAS QRS-wave triggered. The maximum amplitude of the plotted average strain curve during ventricular systole corresponds to the LASr, while LAScd (during passive LV filling) and LASct (during active peak atrial contraction) were derived from the generated curve as already reported (13) and according to the recommended standardization (16) (Figure 1).

### 2.4. Classification of diastolic function

Diastolic function at baseline and by follow-up was categorized by an experienced cardiologist unaware of clinical data, following the ASE/EACVI recommendations (14). We classified diastolic function according to the following criteria: (a) LAVI >34 ml/m^2^; (b) septal e' velocity of <7 cm/s or lateral e' velocity of <10 cm/s, or average e' <9 cm/s; (c) E/e' ratio >14; (d) tricuspid regurgitation peak velocity >2.8 m/s (corresponding to a RV-RA pressure difference >31 mmHg). As previously published (13), women who met 50% of these criteria were considered to present signs of impaired diastolic function (DD1), whereas subjects fulfilling >50% were included in the DD2 group (manifest DD). The rest were graded as DD0 (no signs of diastolic dysfunction).

To classify study participants both at baseline and follow-up, we generated a variable to define progression of diastolic dysfunction (DD_progress). It categorized the sample in women whose DD status worsened and therefore were classified as “progression” (DD0 to DD1 or DD1 to DD2), and in those who prevailed in the same group (stable in DD0 or DD1; classified as “no progression”).

### 2.5. Statistical analysis

Statistical analysis was performed using STATA 14.2. The hypothesis of a normal distribution of phasic LA strain variables was supported by *n* > 30 and tested by Sapiro-Wilk and Levene. Data were represented as mean ± standard deviation for continuous variables and in percentage for categorical variables. For descriptive statistics, we tested the significance of differences in clinical and echocardiographic parameters in subjects with BMI less 25 kg/m^2^ vs. equal to/greater 25 kg/m^2^ using the parametric *t* test for continuous and the non-parametric Chi-Square (*X*^2^) test for categorical variables. A *p*-value <0.05 was considered statistically significant. Finally, to investigate which baseline features of our population were possible determinants of a decline in the LA phasic strain parameters and a worsening of diastolic function, we performed univariate and multivariate regression analysis.

## 3. Results

### 3.1. Study population

The 449 women who qualified for echocardiographic analysis during baseline (13) were reinvited for follow-up examinations. 6.8 years was the mean follow-up time (353.1 weeks; interquartile range 343.1 to 361.1 weeks). Of the 332 participants who replied (73.9% response rate), nine were lost to follow-up, two explorations were cancelled because of other unforeseen medical procedures, and 63 scheduled visits were annulled due to constraints in relation to the SARS-CoV-2 pandemic situation. Hence, 258 participants were included in the BEFRI echocardiography follow-up study. At the time of the examination, we had to exclude two women from the analysis because they had developed moderate to severe mitral regurgitation and atrial fibrillation, and four due to poor acoustic window, resulting in a follow-up sample size of 252 participants (18). Only one of the participants who were excluded due to poor acoustic window presented a BMI ≥25 kg/m². The other three study subjects were excluded due to a foreshortening of the atrial roof in the 4-chamber-view. In addition, we excluded four participants for strain analysis due to inadequate speckle tracking quality of the LA wall (248 women for the follow-up LAS analysis).

### 3.2. Clinical and echocardiographic characteristics

Demographic and echocardiographic characteristics of the total sample analyzed by BMI < or ≥25 kg/m^2^ are represented in Table 1. Of the 252 women, 136 (54%) had a normal BMI (<25 kg/m) at baseline investigation, 103 (33%) were overweight (≥25 kg/m²) and 33 women (13%) were obese (≥30 kg/m^2^). The participants did not show significant weight gain or loss (*Δ*_BMI) during the follow-up. Women with a BMI ≥25 kg/m^2^ were markedly older, had a more frequently history of arterial hypertension and diabetes mellitus type 2, and showed higher levels of low-density lipoprotein (LDL) cholesterol when compared to normal weighted women.

**Table 1:** Clinical and echocardiographic characteristics.

	Total (*n* = 252)	BMI < 25 (*n* = 136)	BMI ≥ 25 (*n* = 116)	*p*-value
Baseline				
Age, years	52 ± 13	47 ± 13	57 ± 12	<0.001
BMI, kg/m^2^	24 ± 4	21 ± 2	28 ± 4	
Waist-to-hip ratio	0.80 ± 0.07	0.78 ± 0.06	0.83 ± 0.07	<0.001
History of arterial hypertension, *n* (%)	61 (24)	15 (11)	46 (40)	<0.001
Diabetes, *n* (%)	12 (5)	3 (2)	9 (8)	<0.001
CAD, *n* (%)	2 (<1)	0 (0)	2 (2)	<0.001
LDL cholesterol, mg/dl	131 ± 37	125 ± 40	138 ± 34	0.004
Creatinine, µmol/L	62.9 ± 9.8	62.9 ± 8.5	62.9 ± 11.1	0.9
BNP, pg/ml	32 ± 26	27 ± 19	38 ± 32	0.002
Systolic BP, mmHg	122.5 ± 14.6	119.2 ± 13	124.2 ± 9.2	0.2
Heart rate, bpm	70.7 ± 10.5	72.2 ± 10	68.9 ± 12	0.6

LAVI, ml/m^2^	29 ± 6	27 ± 5	30 ± 6	0.001
Mitral E wave velocity, m/s	0.74 ± 0.15	0.77 ± 0.16	0.70 ± 0.15	<0.001
e’ average, cm/s	11.3 ± 3.4	12.7 ± 3.1	9.5 ± 2.9	<0.0001
E/e’ ratio	7.0 ± 2.1	6.4 ± 1.8	7.7 ± 2.2	<0.0001
LV-GLS, %	−20.6 ± 2.5	−21.4 ± 2.2	−19.5 ± 2.4	<0.001
LASr, %	39 ± 10	42 ± 9	34 ± 9	<0.001
LAScd, %	−22 ± 10	−26 ± 9	−18 ± 8	<0.001
LASct, %	−18 ± 5	−18 ± 5	−18 ± 6	0.9
Follow-up				
Age, years	58 ± 12	54 ± 13	64 ± 12	<0.001
BMI, kg/m^2^	25 ± 5	22 ± 2	29 ± 5	
Δ_BMI T1 to T2	0.8 ± 1.8	0.7 ± 1.5	0.8 ± 2	0.7
History of arterial hypertension, *n* (%)	79 (31)	21 (15)	58 (50)	<0.001
Diabetes, *n* (%)	17 (7)	5 (4)	12 (10)	<0.001
CAD, *n* (%)	3 (1)	0 (0)	3 (3)	<0.001
BNP, pg/ml	120 ± 118	112 ± 100	129 ± 135	0.3
Systolic BP, mmHg	125.9 ± 11.6	124.8 ± 12	126.2 ± 9.1	0.4
Heart rate, bpm	68.9 ± 10.6	71.2 ± 9.8	67.9 ± 11.2	0.7

LAVI, ml/m^2^	31 ± 7	29 ± 7	33 ± 5	<0.001
Mitral E wave velocity, m/s	0.68 ± 0.15	0.72 ± 0.15	0.65 ± 0.14	0.001
e’ average, cm/s	9.6 ± 3.2	10.1 ± 3.1	8.1 ± 2.6	<0.001
E/e’ ratio	7.1 ± 2.6	7.0 ± 2.1	8.7 ± 2.8	<0.001
DD_progress, *n* (%)	42 (17)	10 (7)	32 (28)	<0.001
LV-GLS, %	−20.8 ± 2.3	−21.4 ± 2.1	−20.0 ± 2.2	<0.001
LASr, %	34 ± 10 *n* = 248	38 ± 9 *n* = 135	30 ± 8 *n* = 113	<0.001
LAScd, %	−18 ± 8 *n* = 248	−22 ± 8 *n* = 135	−14 ± 7 *n* = 113	<0.001
LASct, %	−16 ± 4 *n* = 248	−16 ± 4 *n* = 135	−16 ± 4 *n* = 113	0.7

Data presented as mean ± SD or *n* (%). BMI, body mass index; *Δ*, delta; T1 to T2, time 1 (baseline) to time 2 (follow-up); BP, blood pressure; BNP, brain natriuretic peptide; LAVI, left atrial (LA) volume index; e’, average of septal and lateral early diastolic mitral annulus velocity; DD, diastolic dysfunction; LV-GLS, left ventricular global longitudinal strain; LASr, LA reservoir strain; LAScd, LA conduit strain; LASct, LA contraction strain.

**Figure 2:**
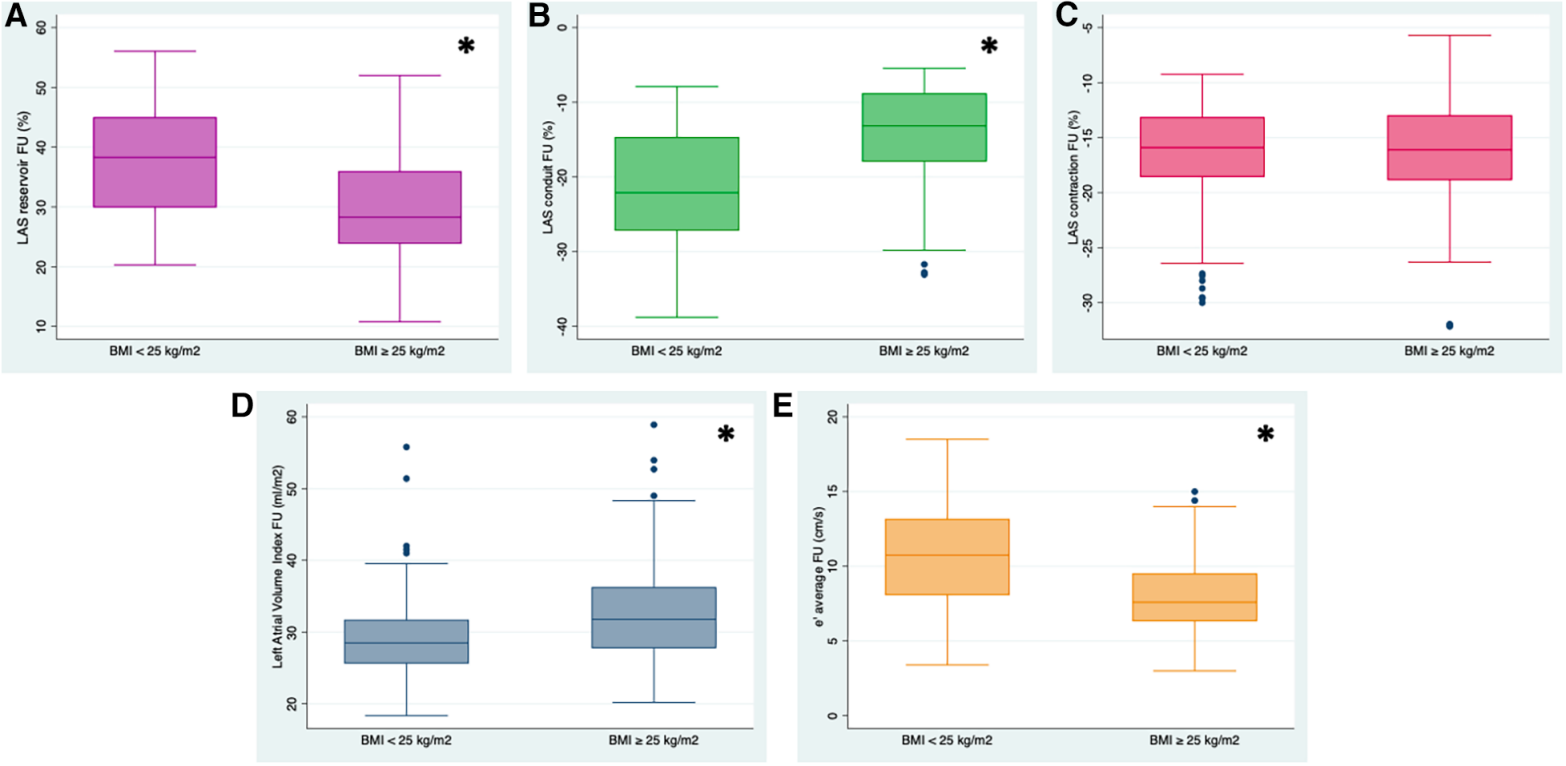
Left atrial phasic strain values in % (reservoir, (**A**) conduit (**B**) contraction (**C**)), e’ average velocity in cm/s (**D**) and left atrial volume index (LAVI) (**E**) at the time of follow-up (FU) in women with BMI <25 kg/m^2^ and BMI ≥25 kg/m^2^. Left atrial reservoir (LASr) was significantly reduced in women with a BMI ≥25 kg/m^2^, who also presented a higher LAVI and decreased e’velocity. Likewise, compared to participants with a BMI in the normal range, LA conduit strain (LAScd) was markedly impaired. *p-value* < 0.01.*

**Figure 3:**
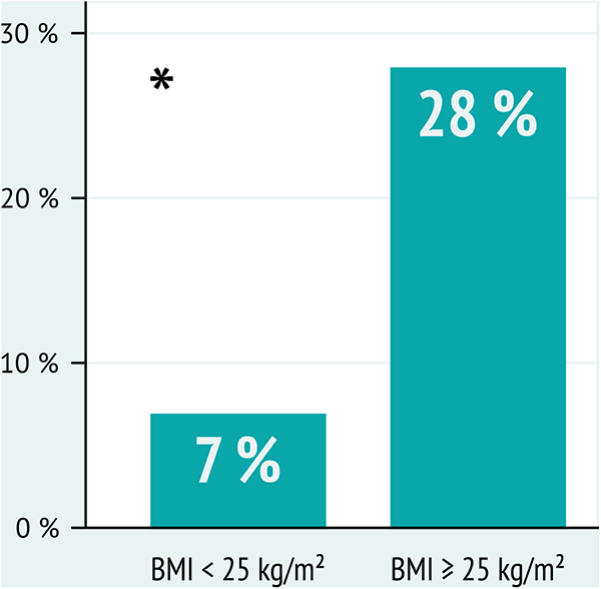
Proportion of women who presented with a deterioration of diastolic function (DD_progress) by the time of follow-up (FU), stratified by body mass index (BMI) at baseline. A significantly higher fraction of women in the group with a BMI ≥25 kg/m^2^ showed a worsening of diastolic function by the time of FU. *p-value* < 0.01.*

Regarding myocardial mechanics, LASr and LAScd as well as LV global longitudinal strain (GLS) were significantly reduced in subjects with a baseline BMI ≥25 kg/m² at the time of baseline investigations and at follow-up (Figure 2). As to classic echocardiographic parameters of DD, women with a BMI ≥25 kg/m^2^ presented higher average LAVI, lower diastolic transmitral inflow E wave and average e' velocities, and greater E/e' ratio by the time of follow-up compared to study participants with a BMI in the normal range (Figure 2). In line with these results, 32 women with a baseline BMI ≥25 kg/m² (28%) showed a deterioration of DD (DD_progress) by the time of follow-up compared with only 10 (7%) of the participants with a baseline BMI <25 kg/m^2^ (Figure 3).

### 3.3. Determinants of LA strain and worsening of DD

Study participants with a BMI ≥25 kg/m² presented a significantly reduced LASr and LAScd both at baseline (13) and follow-up (Table 1), with no significant further worsening of LA mechanics in women with a BMI ≥ 25 kg/m² over time. LASct showed no differences between groups. Age and a BMI ≥25 kg/m² remained significant predictors of LASr and LAScd at the time of follow-up after adjusting for other risk factors, like arterial hypertension, renal function, or diabetes mellitus (Table 2).

**Table 2:** Predictors of diastolic function worsening and left atrial strain at the time of follow-up, multivariate regression analysis.

	Multivariate					
Worsening of diastolic function (DD_progress)	OR	SE	*z*	*p*	95% CI	
BMI	0.991	0.049	−0.181	0.856	0.898	1.089
Creatinine	0.991	0.018	−0.487	0.626	0.955	1.027
HbA1_c_	1.253	0.540	0.417	0.677	0.435	3.669
Hypertension	3.447	0.446	2.777	0.005	1.439	8.325
Diabetes	0.706	0.868	−0.401	0.688	0.120	3.743
Age	1.085	0.020	4.008	<0.001	1.044	1.132
LASr at follow-up	Coeff β	SE	*t*	*p*	95% CI	
BMI	−0.367	0.115	−3.209	0.002	−0.593	−0.142
Creatinine	0.099	0.046	2.153	0.032	0.008	0.190
HbA1_c_	0.270	1.320	0.204	0.838	−2.331	2.870
Hypertension	−1.733	1.227	−1.413	0.159	−4.149	0.684
Diabetes	0.042	2.477	0.017	0.986	−4.837	4.922
Age	−0.405	0.041	−9.847	<0.001	−0.486	−0.324
LAScd at follow-up	Coeff β	SE	*t*	*p*	95% CI	
BMI	0.372	0.086	4.314	<0.001	0.202	0.542
Creatinine	−0.002	0.035	−0.058	0.954	−0.071	0.067
HbA1_c_	−0.353	0.997	−0.354	0.724	−2.317	1.611
Hypertension	1.098	0.939	1.169	0.244	−0.752	2.948
Diabetes	0.354	1.867	0.190	0.850	−3.324	4.032
Age	0.399	0.031	12.790	<0.001	0.337	0.460

OR, odds ratio; β, coefficient; SE, standard error; z, z-value; t, t-value; *p*, *p*-value; LL, lower limit; UL, upper limit; CI, confidence interval; LASr, left atrial reservoir strain; LAScd, left atrial conduit strain; BMI, body mass index.

When analyzing independent risk factors of DD worsening in the follow-up, BMI showed no significant association in a multivariate logistic regression, as age and arterial hypertension appeared as stronger determinants.

## 4. Discussion

In this prospective longitudinal study, we assessed the impact of overweight and obesity on phasic LAS and diastolic function in women after a mean follow-up period of 6.8 years in women. We found that a BMI ≥25 kg/m² was significantly associated with reduced LAS values and with a markedly higher rate of diastolic function worsening compared to participants with a BMI <25 kg/m^2^.

Overweight and obesity are well-known risk factors for different cardiovascular pathologies, such as atrial fibrillation (AF) and heart failure with preserved ejection fraction (HFpEF) (19–21). However, there are conflicting reports (3) and the data seem unclear, as a paradoxical protective effect of obesity on heart failure has also been discussed (4).

A high BMI is associated with an augmented risk of DD as shown by Rozenbaum et al. (1). The investigators reported that metabolically healthy overweight and obese patients were more likely to present abnormalities in most echocardiographic parameters recommended by the ASE/EACVI for the assessment of DD than patients with a BMI <25 mg/kg^2^, including LAVI >34 ml/m^2^. While on one side, phasic LAS impairment has been widely linked to DD, even without LA volume enlargement (6, 7, 22), on the other side Shemirani et al. (3) found no relationship between a high BMI and LVDD in a young obese population without other comorbidities. In our cohort of women with a low cardiovascular risk, a significantly greater number of overweight and obese subjects showed a worsening of diastolic function at the time of follow-up compared to the group with a BMI <25 mg/kg^2^ (28% vs. 7%), still presenting a mean LAVI <34 ml/m^2^ but notably reduced phasic LAS values. However, it was age and arterial hypertension which appeared as stronger determinants for diastolic function to be worsening over time, as described in other larger cohorts (1). Very recent data suggest that the use of alternative indices to BMI that do not incorporate weight, as for example waist-to-height ratio, may reflect better the adiposity linked to a higher cardiovascular risk, and therefore be more adequate for stratification (23).

Current guidelines (14) recommend the use of LAVI to diagnose and stratify DD because of its widely recognized role in cardiovascular risk stratification (24, 25). Nevertheless, there is increasing evidence on the relevance of the additional assessment of LA function for detection and classification of DD (5), given that it frequently shows alterations prior to LA enlargement (6, 7, 13). Furthermore, the growing interest about LA function relies on its prognostic value in many cardiovascular conditions (26). As already described in the BEFRI trial and as recently published (18), reduced phasic LAS entails predictive value regarding the worsening of diastolic function over time.

The assessment of LA anatomy and function using alternative imaging techniques, such as cardiac MRI, has become a suitable and consistent method over the last few years (27, 28) and needs to be considered in addition to echocardiography, especially to avoid its limitations, such as foreshortening of the atrial roof and the poor quality of the images due to bad acoustic windows, which is a common issue in overweight and obese population. The analysis of myocardial deformation using different quantitative tracking techniques as feature tracking (CMR-FT) (29) or fast strain encoded magnetic resonance (fast-SENC) (30) is growing in importance and has shown prognostic impact in stratifying population in risk of cardiovascular events as heart failure (31, 32) or atrial fibrillation (33). Due to the good visualization of the LA wall, quantifying LA volumes and strain using MRI may be advantageous for risk classification and evaluating response to therapeutic measurements in obese to assure reproducibility and accurate tracking. Nevertheless, cardiac MRI is still not available in every clinical setting and is not always feasible in terms of cost-efficiency, mobility, etc., when follow-up examinations are needed. Something relevant to consider since reference values are technique and vendor specific (28, 34).

For the use of LAS in clinical practice using echocardiography, normative data have been thoroughly reported (35), most recently in a large cohort of healthy individuals (36). Moreover, concerning clinical implications, the assessment of independent determinants of phasic LAS is important for its adequate interpretation. Sugimoto et al. described a mean value of 42.5% (interquartile range 36.1–48.0) regarding LASr; −25.7% (−20.4–31.8) for LAScd; and 16.3% (12.9–19.5) for LASct in healthy subjects, consistent with the data that we present in our cohort of women with BMI <25 mg/kg^2^. In accordance with our results, Chirinos et al. (11) published a significant lower amount of LASr and LAScd in a large cohort of overweight and obese persons aged 35–55 years.

The positive effects of weight reduction on myocardial mechanics have already been reported in metabolically healthy obese patients who undergo bariatric surgery (37, 38, 39), indicating the potential reversibility of impaired LA function. In the same line, the beneficial clinical impact of drug-induced weight reduction using semaglutide in patients with already diagnosed heart failure with preserved ejection fraction, as recently published by the authors of the STEP-HFpEF Trial (40), highlights the importance of routinely implementing early cardiovascular risk detection tools such as myocardial strain, especially from the point of view of addressing primary prevention measures.

### 4.1. Limitations

Our study holds several limitations. (A) Mainly due to the complicated health situation resulting from the SARS-CoV-2 pandemic, 72 women initially enrolled were lost to follow-up with potential impact on the power of our study. Our results should be confirmed in larger prospective trials, also considering possible sex-related differences. (B) The assessment of the BMI represents an indirect measurement to classify obesity with limited diagnostic value to assess body fat composition and its distribution, which play a crucial role in lipid and glucose metabolism as well as in low grade inflammation (41). Direct quantification of body fat with for example energy x-ray absorptiometry may have added value to the study. Nevertheless, estimating body fat by assessing the BMI, the waist circumference, or the waist-to-hip ratio, as described in our data, are easily obtained, and are widely used in clinical routine. BMI still represents the most frequently used parameter of obesity and shows, in contrast to the parameters waist-to-hip ratio or waist circumference, consistent cut-offs in many studies (42). (C) We did not stratify the results by age, although its influence on DD and on BMI itself is well-known (43, 44). Moreover, all phases of LAS diminish with age as shown in the NORRE study (36). In the multivariate analysis, however, BMI remained significantly associated with a worse LAS at follow-up after adjusting for age and other risk factors. On the other hand, age and arterial hypertension appeared as stronger determinants of DD worsening than BMI. (D) We only examined women, a fact that relates to the initial design of the BEFRI trial (12). Nevertheless, no relevant gender differences in LAS parameters have been described to our knowledge (35, 36). (E) Specific hormonal status was not assessed in our study cohort, a limitation that should be considered given the well-known association of postmenopausal hormonal changes with cardiovascular risk in women (45). (F) A dedicated LAS-tracking software has been recently implemented in clinical practice, improving measurement reproducibility (46). At the time of baseline investigations, this tool was not available, and therefore we used the LV-dedicated strain software for both exploration time points, as the use of the same analysis software is recommended for follow-up measurements (16), and no significant differences in mean LAS values have been described comparing both methods. (G) Our study did not aim to determine the superiority of the LAS values when compared to conventional diastolic dysfunction parameters. The LAS measurements remain an added value and should be used and interpreted in addition to the standard echocardiographic parameters. The superior value of LAS is to be determined in larger prospective trials, which include a more heterogeneous female and male study population.

## 5. Conclusion

Our data demonstrate significant reductions in phasic LAS after a mean follow-up time of 6.8 years in women who initially presented with a BMI ≥25 kg/m^2^. In addition, overweight and obese women showed a higher rate of worsening diastolic function compared to study participants with a BMI <25 kg/m^2^. Our data suggest that overweight and obesity may significantly impair myocardial function in women already after a medium-term follow-up period. However, these findings need to be confirmed in larger prospective trials that include men and women and that investigate the relationship between BMI, LAS and the deterioration of diastolic function over time and the development of heart failure signs and symptoms.

## Data Availability

The original contributions presented in the study are included in the article/Supplementary Materials, further inquiries can be directed to the corresponding authors.
